# Commentary about mesenchymal stem cells and scarred vocal folds

**DOI:** 10.1186/s13287-020-01693-9

**Published:** 2020-05-07

**Authors:** Alexia Mattei, Jérémy Magalon, Mélanie Velier, Françoise Dignat-George, Antoine Giovanni, Florence Sabatier

**Affiliations:** 1 Department of Oto-Rhino-Laryngology and Head and Neck Surgery, APHM, Assistance Publique-Hôpitaux de Marseille, La Conception University Hospital, 147 bd baille, 13385 Marseille Cedex, France; 2 Aix Marseille Univ, CNRS, LPL, Aix-en-Provence, France; 3 Therapy Department, Aix Marseille Univ, APHM, INSERM, CBT-1409, La Conception University Hospital, Cell, Marseille, France; 4 Aix Marseille Univ, INSERM, INRA, C2VN, Marseille, France

**Keywords:** Vocal folds, Scarring, Fibrosis, Mesenchymal stromal cells, Glottic cancer, Bone marrow

## Abstract

A commentary to “Hertegård, S., Nagubothu, S.R., Malmström, E. et al. Treatment of vocal fold scarring with autologous bone marrow-derived human mesenchymal stromal cells - first phase I/II human clinical study. Stem Cell Res Ther 11, 128 (2020)” concerning the surgical intervention including a scar resection, the use of the Voice Handicap Index, the surgical and regulatory points of view regarding the inclusion of patients with laryngeal carcinomas history, and the side effects of bone marrow harvesting.

## Main text

We have read with great interest the article by Hertegård et al. entitled “Treatment of vocal fold scarring with autologous bone marrow-derived human mesenchymal stromal cells - first phase I / II human clinical study” [1]. We would like to congratulate the authors of this clinical trial which highlights the anti-fibrotic potential of cell-based therapy using mesenchymal stem cells (MSC) and brings hope in the difficult treatment of scar vocal folds. However, we would like to discuss several points that have drawn our attention:

1. Regarding the surgical intervention, we are surprised by the choice of scar resection before injection step. This raises two major concerns from our point of view. First, it introduces a significant bias in the interpretation of preliminary efficacy results. Indeed, resection of scar tissue is one of the potential treatments offered in this type of disease. Although its efficacy is known to be limited, we cannot exclude that improvements observed in this study could result from this resection. Conversely, resection of scar tissue could increase the tissue defect and therefore potentially the glottic air leakage. This surgery can cause an aggravation of dysphonia [2], as observed in their animal model [3] and specified by the authors in the introduction [1]. This is also the reason why the authors explain that they did not perform a control arm with scar resection alone. We notice in the clinical study that the Voice Handicap Index worsens in 6 patients out of 16. An aggravation of the vocal folds’ vibration is also noted in 4 patients as this is analyzed in the discussion. The encouraging results of this study could therefore have been even better if the MSC have been injected into scar tissue without prior resection.

2. Regarding the Voice Handicap Index (VHI) which is considered as the most important parameter to report patient’s voice satisfaction, we would like to remind the authors that a change in the VHI score is considered as clinically significant if equal or greater than 18 points and not 13 as mentioned in the manuscript [4].

3. From a regulatory point of view, the authors excluded patients with active laryngeal neoplasia but included patients with a history of laryngeal neoplasia, without specifying the time required between recovery from this cancer and injection of MSC. We would like to know what studies the authors and regulatory agencies referred to for authorizing the injection of MSC into a scar from a past-cancerous lesion. Indeed, the impact of MSC on tumor evolution and related mechanisms is still under discussion although recent studies seem in favor of a good safety profile in this context [5, 6].

4. From a surgical point of view, laryngeal carcinomas (laser endoscopic cordectomy) are the largest provider of vocal folds scars, much more than the scars induced by the treatment of benign vocal lesions or congenital sulci. This is due to the fact that the scars induced during initial removing surgery are often much deeper, sometimes involving the thyroarytenoid muscle in its entirety [7] and creating fibrosis. Thus, in these cases, the main problem is the important tissue defect causing a major glottic air leakage and it is obvious that an anti-fibrotic therapy may not be sufficient. A medialization of the vocal fold by volumizing injection is therefore necessary in addition [2]. Surprisingly, the authors added hyaluronic acid as a scaffold in half of the 16 patients without considering the size of defect. Combined to the point 3, we raise the relevance to include these large defects in this type of study since the vibrational disorders are secondary.

5. The author did not develop the side effects induced by bone marrow harvesting from the iliac crest. Regarding this point, we reflect upon the duration of post-operative pain which could be balanced using other sources of MSC whose removal is potentially less invasive (including adipose tissue).

In conclusion, cell therapies are emerging as promising strategies in scarred vocal folds. Future controlled and comparative studies are now expected to confirm efficacy and identify the optimal cell source. A better selection of the indication and refined knowledge of the different mechanisms underlying the clinical efficacy are still expected.

## Data Availability

Not applicable

## Abbreviations

MSC: Mesenchymal stem cells
VHI: Voice Handicap Index
